# Adequacy of care provision in long‐term home nursing arrangements: A triangulation of three perspectives

**DOI:** 10.1002/nop2.548

**Published:** 2020-07-02

**Authors:** Anna‐Henrikje Seidlein, Maresa Buchholz, Sabine Salloch, Ines Buchholz

**Affiliations:** ^1^ Institute of Ethics and History of Medicine University Medicine Greifswald Greifswald Germany; ^2^ Department Methods Institute for Community Medicine University Medicine Greifswald Greifswald Germany

**Keywords:** caregiving, home nursing, informal care, long‐term home care, qualitative research, social inequality, underprovision

## Abstract

**Background:**

A growing proportion of older people in Germany receive long‐term care from informal and professional caregivers at home. Their personal assessment of the individual care situation is scarcely considered.

**Aim:**

This study aimed to explore the subjective views of care recipients, informal and professional caregivers on the adequacy of care provision in long‐term home care arrangements.

**Design and Methods:**

Qualitative semi‐structured face‐to‐face interviews were conducted with ten care recipients, ten professional caregivers and eight informal caregivers to capture their perspectives on the adequacy of the care received and delivered. Qualitative content analysis was applied using MAXQDA software.

**Results:**

All groups highlighted that they perceive an *underprovision* of care, even though their explanations differed. The underprovision was mainly described regarding the quality rather than quantity of services. It occurs especially in interpersonal relationships and social inclusion, where the gap between the self‐perceived current situation and the desires of those affected is most prominent. The ambivalent impact of home care on social participation becomes apparent. Perceptions of an *overprovision* of care range from the view that it appears mainly with respect to informal care to the statement that it is currently non‐existent or generally impossible. *Misprovision* of care is experienced as serious whenever the interviewees face the challenge of preserving existing abilities or regaining certain skills.

## 1 BACKGROUND

As life expectancy rises and chronic diseases accumulate, the likelihood of long‐term assistance from others' also increases (Schnitzer et al., 2019). In 2017, 3.41 million Germans were in need of long‐term care (LTC) (German Federal Statistical Office, 2018) and this high proportion is still rising; 81% of these care recipients were 65 years and older and 35% were at least 85 years old. Most of them are cared for by informal caregivers—“a person who provides unpaid care to someone with a long‐term illness, disability or other long‐lasting health or care need, outside a professional or formal framework” (Eurocarers & European Cancer Patients Coalition, 2017, p. 10)—or receive both formal and informal care at home (German Federal Statistical Office, 2018, p. 5). Thus, at least three groups are directly involved in long‐term home care (LTHC): informal caregivers (ICs), professional caregivers (PCs) and care recipients (CRs). Their situation is characterized by different interests and partly conflicting wishes, expectations and needs that reflect juxtaposed realities.

## 2 INTRODUCTION

For ICs, care takeover influences all aspects of their daily life (Bauer & Sousa‐Poza, 2015) and is accompanied by serious changes in their own lifestyle for a considerable period of time (DAK, 2015). It may also negatively affect their own health (Kaschowitz & Lazarevic, 2020; Zwar, Konig, & Hajek, 2018): ICs can feel overburdened, might suffer from loneliness (Vasileiou et al., 2017), depression, anxiety (Woodford, Farrand, Watkins, & LLewellyn, 2018) and multiple other “unseen costs” (Jowsey, Strazdins, & Yen, 2016). In addition, the mean age of ICs increases and therewith the likelihood of own health impairments present before care takeover.

Compared with other European countries, the situation of PCs—especially geriatric nurses (a large share of nurses working in LTHC hold a specialization in geriatric nursing; home health nursing is not a specialization for nurses in Germany)—in Germany is characterized by the fact that they receive less appreciation of their demanding work with, at the same, less attractive working conditions (Theobald, Szebehely, & Preuß, 2013). Moreover, nursing in Germany is also distinguished by the low share of academization and uneven education (Appendix S1). This contributes to the fact that especially geriatric nurses often leave the profession after a few years (Wendsche, Hacker, Wegge, & Rudolf, 2016).

The developments described need to be considered in the light of the compulsory German long‐term care insurance (LTCI), which is designed as partial coverage insurance (European Observatory on Health Systems & Policies, 2016). Guided by the principle of subsidiarity, the LTCI's principle of “home nursing before nursing home” becomes increasingly problematic given the fact that societal and familial circumstances have changed since its implementation in 1995 (Theobald & Luppi, 2018). The situation delineated above is aggravated in sparsely populated, economically weak regions of Germany, such as Mecklenburg‐Western Pomerania, which is particularly affected by demographic change and care dependency.

Although an extensive body of literature exists on the growing need of older people for LTC in their home environment, data reflecting the evaluation of LTCI coverage and access to affiliated financial and personal aids as experienced by those affected remain scarce (Scheil‐Adlung, 2015). This neglect ignores potential underprovision, over‐ and misprovision of care and their negative impact on individual and societal level. Underprovision is defined as “Failure to deliver a service that is highly likely to improve the quality or quantity of life” (Elshaug et al., 2017, p. 192). In the nursing context, it is referred to in the special appearance of “missed care”/“care left undone”/”unfinished nursing care.” Overprovision refers to “a service that is unlikely to increase the quality or quantity of life” (Elshaug et al., 2017, p. 192). Regarding nursing care, this phenomenon is even harder to determine, as it is linked to the question of what is considered as nursing care and thus which tasks are or should be part of comprehensive nursing care. Misprovision occurs when “irrespective of the level of the objective need […] health care services are not correctly provided or are not provided in the quality required” (Advisory Council on the Assessment of Developments in the Health Care System, 2014, p. 15). Only a few attempts have been made to operationalize and measure the over‐ and underprovision of care in LTHC (Lahmann, Suhr, Kuntz, & Kottner, 2015). To date, there has been limited research regarding the subjective perspectives of the relevant parties directly involved in caregiving at home. The present article reports CR, IC and PC perceptions concerning the adequacy of the care received and delivered in LTHC arrangements. These results are part of a larger study on the lived experience in LTHC arrangements. The other empirical results are published in (Seidlein, Buchholz, Buchholz, & Salloch, 2019a, 2019b).

## 3 METHODOLOGY

A qualitative approach was chosen to gain deeper insight in CRs', ICs' and PCs' views of enabling and performing, as well as receiving care at home. One of the main research questions studied was How do CRs perceive the current care situation in their LTHC arrangement? How do ICs and PCs perceive the current care situation in the LTHC arrangement(s) they are engaged in? A convenience sample was recruited via three home care services, covering rural and urban regions of the federal state of Mecklenburg‐Western Pomerania in north‐eastern Germany. Criteria for study participation are shown in Table 1. Nursing management staff of home nursing services approached potential interview partners and passed contact information on to the research team if permitted. Written informed consent was obtained from each participant. The study was approved by the institutional review board of the University Medicine Greifswald (BB 123/16).

**TABLE 1:** Inclusion criteria for study participation. Reprint from Seidlein, Buchholz, Buchholz, and Salloch (2019b)

	Informal caregivers	Care recipients	Professional caregivers
Inclusion criteria	providing long‐term home care for relevant others (parents, partner) cooperation with professional caregivers	recipients of long‐term home care support of formal caregivers (nursing service) *and* informal caregivers (e.g. family members)	registered nurses working in home care services cooperation with informal caregivers

Semi‐structured face‐to‐face interviews (Appendix S2) were conducted between October and December 2016 by AHS and two graduate students trained and supervised by the research team. The interviews were carried out at each participant's preferred location (at home, at the workplace, at the researcher's office). Five interviews were conducted in attendance of the interviewees' significant other. Interviewees were asked to report on the care situation in home nursing arrangements, respectively, about how sufficient he/she perceives the help given or received at the moment, whether he/she feels well cared for and, in case if not, for what reason. The interviews were audio‐recorded and transcribed verbatim. After transcripts have been read and re‐read to engender familiarization with each interview, the principles of qualitative content analysis, according to Mayring (2014, p. 53ff.), using MAXQDA 12 software have been applied. Data analysis was driven deductively (questions from the interview guide) and inductively (new themes raised by the interviewees). Each interview was coded line by line by two researchers (AS and MB, or AS and IB). Codings (coded text passages) and categories (denomination of the coded passages) were reviewed for (dis‐)similarities to result in a consented form for the next interview and so forth. Unclear passages were discussed in the multidisciplinary research group (AS, IB, MB, SS) until consensus was reached.

## 4 RESULTS

None of the persons approached declined to participate in the study. The sample (Table 2) consisted of *N* = 10 CRs (mean age: 80.7 years) affected by multiple chronic conditions and receiving LTC at home by ICs (*N* = 8) and PCs (*N* = 10).

**TABLE 2:** Sample characteristics. Reprint from Seidlein et al. (2019b)

	Informal caregivers	Care recipients	Professional caregivers
	(*N* = 8)	(*N* = 10)	(*N* = 10)
Sex			
Male	1	4	3
Female	7	6	7
Age			
Mean ± *SD*	67.1 ± 15.5	80.7 ± 9.6	35.8 ± 7.5
(range) in years	(37–87)	(62–95)	(28–49)
Marital status			
Single	0	1	3
Married	8	4	6
Divorced	0	0	1
Widowed	0	5	0
Children			
0	1	2	—
1	1	3	6
2	3	4	4
3	2	1	—
4	1	—	—
Formal education			
9 years	4	6	—
10 years (middle school)	2	3	8
12 years (high‐school diploma)	2	1	2
Length provision informal care			
Mean ± *SD* (range) in years	5.3 ± 4.4 (1.5–13)		
Professional experience			
Mean ± *SD* (range) in years			12.8 ± 6.7 (3–22)

The interviews lasted between 16 and 134 min, with an average of 48 min each. The analysis revealed two themes: first, the “LTCI system as a barrier to care” with the three sub‐themes “unrealistic assessment instrument,” “obscure assessment process,” “Individuals' competence”; and second, “Care overlooking individual needs” with the three sub‐themes “overprovision,” “underprovision” and “misprovision.”

### 4.1 LTCI SYSTEM AS A BARRIER TO CARE

Access to statutory benefits depends on the definition and assessment of the being “in need of care” in terms of the German Social Security Code XI. This theme summarizes the problems depicted by the interviewees regarding the current design and implementation of the LTCI.

#### 4.1.1 Unrealistic assessment instrument

Informal caregivers described the current assessment instrument to determine care dependency as escapist, “construed by someone who has never cared for anybody” (PA13) and resulting in inadequate allowance. Counting the time needed for single actions (such as getting dressed) leads to a delusive “Minute Care” that does not reflect the true needs. The PCs agreed with that, explaining that some older people in need of a Care Level due to specific restrictions do not get it. From the PCs point of view, the criteria are oriented towards symptoms and concentrate on deficits instead of searching for causes. The focus on bodily needs and the failure to take subjective feelings and needs into account was also stressed by the CRs. PCs summed up that the Care Levels are insufficiently differentiated and that many of the older persons do not get the proper one. As a result, they must pay extra because their Care Level does not cover the costs.

#### 4.1.2 Obscure assessment process

The assessment process was criticized as protracted and non‐transparent by all three groups. PCs stressed that the assessment represents only a snapshot—where the CRs also exhaust themselves to show what they are still able to do—and that, instead, there should be more appointments to assess the care needs more realistically. In their opinion, the assessment “unfortunately depends very, very much on the evaluator” (PP8) and, thus, they saw great differences in the quality of the assessment process (e.g. observation and questioning of the CR) and in the results, where they often perceived misguided judgements. For this reason, the PCs try to be present during the assessment as they know on what the result depends. They also reported on prolonged processes and the necessity of entering multiple objections to get a Care Level.

#### 4.1.3 Individuals' competence

Bureaucratic obstacles were described as a major concern and were also seen as one reason for underprovision as those affected cannot overcome them. Some ICs reported that they did not understand how the system works and that for them as laypersons it is not possible to assess the services' adequacy. CRs also stressed that knowing the system and people working in the insurance companies was helpful for them and facilitated access to aids and therapeutic appliances. The insurance company's authority to decide about care aids (equipment) was mentioned as incomprehensible, not only because it takes too much time, but also that the clerks do not have the expertise to decide on the needs of CRs and ICs. The PCs in addition challenged the medical knowledge and nursing competence of some evaluators.

All groups highlighted that they set their hope in the forthcoming five “Care Grades.” This should affect the current care situation positively as it announces a system that goes beyond physical needs and focuses on the mental and social dimensions of care needs.

### 4.2 CARE OVERLOOKING INDIVIDUAL NEEDS

The evaluation of the care provided varied not only in the views of the different groups, but also with respect to the different aspects of daily life. This theme depicts appearances over‐, under‐ and misprovision of care and factors contributing to them.

#### 4.2.1 Overprovision of care

All ICs negated the existence of an overprovision of care, whereas PCs view on overprovision were divided: For some professionals, overprovision is impossible as “there can never be too much care” (PP9). Others did not observe it in their working environment, whereas yet others described how overprovision of care occurs in their daily routines. In this context, they mentioned situations where:

Some individuals are able to do more than they actually do. And sometimes I don't understand why we are in this household, because there are patients where I do the laundry, shave, but the man could do this on his own. Or I do the personal hygiene in bed and afterwards, the man stands up and goes into the kitchen. That makes me think ‘Oh my god, he can walk, he has two well‐functioning hands, he could wash his face or genital area by himself’. (PP3)

In their opinion, overprovision of care exists due to personal and system reasons. Regarding personal reasons, there are nurses described who show a “helper syndrome,” taking everything out of the CRs' hands regardless if he or she is able to do it by himself or herself. Consequently, CRs quickly get used to it and delegate everything. On the other hand, there are CRs who can do more, but do not want to and demand that everything is done by the professionals right from the beginning. They are described as “lazy” (PP5) and “like to be indulged” (PP8). They let themselves go and even use different strategies to convince the professionals that they need more assistance or play the professionals off against each other to make them do what they want. At the system level, financial disincentives lead to offers of services and “showering CRs with services” (PP7) that are not necessarily needed because the parties involved strive for full use of the Care Level awarded by the LTCI.

Overprovision of care is non‐existent for all CRs with respect to professional nursing but occurred regarding ICs due to “too much” solicitude. They described that their relatives did not know or did not understand what they are still able to do in daily life and, therefore, feel overprotected when relatives take away tasks that they could still do by themselves. CRs do not necessarily mention this subjective feeling of overprotection and overprovision of care to their ICs as they do not want to “affront” them and are afraid to hurt them.

#### 4.2.2 Underprovision of care

The ICs complained about a wide range of underprovision of care. This comprised mainly personal aid for them and the provision of care equipment. They particularly stressed that there is less incontinence material provided than is needed, which they described as “a really big mess (…) The insurance company recently pays only 24.99 € monthly, but you cannot look after someone with this amount of money. This is beneath contempt. This is contrary to fundamental human rights” (PA2).

The PCs highlighted that underprovision of care occurs in different aspects of daily life, but is especially regarding the following: first, special equipment (e.g. lift, toilet chair) that would be necessary but that is either not provided because the general practitioner does not prescribe it, or if so, the insurance does not pay for it (e.g. because the Care Level does not cover this). In both cases, the CR cannot pay for the equipment needed by himself/herself. Second, a lack of transportation services to keep appointments for diagnostic and therapeutic procedures or just to have a chance to get out. Third, support for ICs (e.g. offers to relief them from the burden of care work) and fourth, time for high‐quality care. Besides the technical and medical actions the PCs have to perform, scarcities of communication and social participation are considered as problematic: “You go in, do your work that you have to do and leave” (PP5). Nevertheless, their view concerning the reasons for underprovision were divided. Some PCs stated that the extent of support is sufficient regarding the services requested, but that the usage pattern of CRs and ICs shows great variations. They make the CRs as “service users” responsible for their experiences of not being supplied sufficiently. According to the PCs, one possible cause of the appertained benefit not being used is that the CRs do not want to accept that they are in need of care, that is, they do not want to accept the real severity of their care needs and in the case that he or she does not accept care, no suitable care can be provided. These CRs waive the support that would be necessary from the professionals' point of view because they try to keep the sovereignty they are afraid of losing:

Some individuals don't want that, because they insist on their self‐reliance. Bizarrely, those who are to a great extent in need of our care, refuse it. (…) I know for sure that, for example, one woman is not able to take off her shoes; she goes to bed with her shoes on. The money is there, I could help her, also in the evening, get her ready for the night, but she refuses. (PP6)

Home nursing services can offer a broad range of services but not all of them are being used. One nurse, thus, concluded “Everything is there, one just has to ask for it” (PP2). Furthermore, home nursing services “can offer everything” (PP2), but the professionals described different degrees of willingness to expend (extra) costs for professional care. Some individuals do not want to spend their money for care they would need in the eyes of the PCs. Hence, these professionals identified one main reason for the underprovision of care as lying in the older people's different manners of use and willingness to make an additional payment for care services:

It would be better for some individuals to buy more services, but that is their problem, not ours. I think, we perform our services really sufficiently, but sometimes the services booked are not enough. They would have to buy more services from the nursing service or exhaust all the possibilities the Care Level gives them. (PP1)

Other professionals state that systematic underprovision of care occurs as a result of misconception in the LTCI system (assessment and classification system for care dependency). PCs recognize that the financial and social situation of the CRs and the limited financial coverage by the LTCI are also reasons for underprovision, as some families cannot raise the money for extra services not covered by the LTCI. These nurses raised the idea of a fully comprehensive LTCI.

On the other hand, they added that CRs and their ICs do not use all the sources of financial support they could, because they do not want to unveil their financial situation:

But they only have themselves to blame, because nowadays, they can get every care aid; they don't have to relinquish anything. No one has to lie in bed, no one has to forgo fresh air, everyone can be brought outside somehow, sometimes more and sometimes less complicatedly (…). The only thing you have to do is make an application, for example, at the Social Welfare Office, you just have to justify it. That can be a long process, but nothing is impossible. (PP2)

Some professionals described that they are aware of the older peoples' needs and reported on extra efforts, not being paid for, to meet their wishes and to compensate shortcomings: “Many of my colleagues visit them [the CRs] even after work, they take their free time without looking at the clock, go shopping for them, go for a walk with them and so on, because they also establish a close relationship with the patient” (PP7).

Underprovision of care for CRs is experienced most concerning interpersonal relationships. The CRs suffer from a reduction of care to physical needs, such as bodily care. Communication (e.g. in the PC–CR relationship) and social inclusion are disregarded. Another main neglected area comprised mobility, a point that is strongly interwoven with owning care aids and appliances that facilitate mobility but can also hinder it if they are missing or do not function faultlessly. Thus, for CRs, not only mobility enables social participation, but their social contacts allow mobility. One CR explains that “If I had a lot of money, I would buy a car which I could get into with my wheelchair (…) Old and sick, that's what you shouldn't get here. But as long as I still have my friend; he takes me on a trip sometimes” (PB2). Mobility is the precondition to adhere to therapeutic appointments and CRs bewail not having the possibility to get there other than by using a taxi that they have to pay for on their own without any support from the insurance company. Moreover, CRs indicate that they receive less physio‐ and ergotherapy than they need to retain their abilities effectively. They see reasons for that in the pressure on the nurses to perform and extra payment for services, for example, bathing instead of washing. The CRs do not mention any strategies to compensate for their subjective underprovision in these fields.

#### 4.2.3 Misprovision of care

Interviewees also mentioned several fields where they perceive avoidable harm for the CRs due to the work not being done properly (e.g. regarding standards of professional practice).

Firstly, the CRs bring up an imbalance of the cost‐benefit ratio, for example regarding additional therapies that aim at retaining physical abilities and independence. One CR reported her experiences concerning prevention and rehabilitation: “Once they also prescribed speech therapists, but the circumstances, getting ready and everything, the waiting time, to get an appointment, they are more negative than the positive effects of the therapy” (PB1). Furthermore, the CRs stress that the care is quantitatively provided but lacks high quality. They also reported that their personal expertise is not taken into account, which is not only frustrating for them, as they not only perceive themselves as experts on their situation and needs, but this practice also ignores the usage of precious resources inside the CR.

The ICs described the care products provided as being inadequate for their needs: “I constantly buy disposable nappies because they are not included in the program, but they send me so many bed undersheets I don't need, that I can wallpaper my flat with them” (PA13). Furthermore, they indicated the missing and/or not strict enough monitoring of the home nursing services. The official quality reports seem to be susceptible to manipulation, as they experience that not all criteria are met, while nearly all home care services get a good rating. They have learnt that “it does not mean anything if someone calls himself/herself a nursing professional. That does not mean that he/she is able to care for other human beings” (PA2). They described their experiences of what they perceive as professional misbehaviour, which is mainly missing empathy, as reflected in what an IC told a PC when she perceived such behaviour: “You are not sorting screwdrivers here, but you are working with human beings and they are dependent on you and your good care and if you are not able to do that you are in the wrong job” (PA2). They also observe mistakes and mishaps by professionals that might lead to harm for the CR. Finally, not only professionals but also unqualified personal (e.g. home helps) are described as taking over nursing care duties.

## 5 DISCUSSION

All interviewees reported serious problems with the assessment tools and processes as well as with over‐, under‐ and misprovision of care in their various manifestations. When interpreting the results, it should be noted that this study was carried out at the time of the implementation of a healthcare reform (Appendix S1), which explains the hopes placed in the system change by the interviewees. Even though the individuals affected represented in our sample have placed high expectations in the amendment, first data have shown that the claiming of benefits and the quality remain insufficient even after the latest reforms (Eggert, Storch, & Sulmann, 2018).

The study results suggest that care needs experienced subjectively and those objectively assessed and adjudged on a statutory basis do not necessarily match. Furthermore, professionals might assess and weigh the care needs in a different way compared with the common assessment instruments. Our findings thus confirm other study results (Bergmann & Brühl, 2017; Lipszyc, Sail, & Xavier, 2012, p. 24). Moreover, the subjective need for LTC does not lead inevitably to a formal demand and, thus, provision of care. It is known that individuals with lower social status are more likely to be exclusively informally cared for and to receive fewer support, while more frequently applying for care allowances (Kruse & Schmitt, 2016). This fact points to one aspect of social inequality in LTHC (Möller, Osterfeld, & Büscher, 2013) that not only affects PCs, who are confronted with an unbalanced situation between effort and reward, but also ICs. Accessibility of benefits remains a major challenge that could not be covered by our study due to the recruitment of interviewees via home nursing services. Those CRs and ICs without any formal support might have brought up other problems. Not least, availability is increasingly problematic and leads to provisional gaps. Even if services are adjudged through LTCI, they cannot always be used as they are not offered everywhere and at any time. Such geographical disparities influencing the LTHC arrangement differ between various regions in Germany, are well documented (DG Employment Social Affairs & Inclusion, 2018) but still receive little attention (Ilinca, Rodrigues, & Schmidt, 2017). As a result, what a CR and his/her ICs receive does not necessarily correspond with what they are entitled to. The importance of the respective understanding of what nursing care is, what its goals are and what its responsibilities include is also directly evident in other aspects of underprovision: what constitutes missed care for some professionals is not part of their core competencies and professional duties for others, instead it might be even an act of supererogation for them. However, even if they see it as their responsibility to fulfil certain tasks, they still need to prioritize their actions under the current working conditions. The criteria they use to make these decisions, that might also entail implicit rationing, should be subject to further empirical ethics research in this setting.

Social inequality in LTHC is further fostered by present problem‐solving strategies concerning social participation. Although the latter has been widely unattended until now, it is highly relevant, as shortcomings in the supply for CRs occur especially in aspects which can be bought as extra services. Regarding this provisional gap, social inequality becomes most precarious. A private market has developed, for example for 24‐hr live‐in arrangements with caregivers from Eastern Europe, that facilitates exploitation in one's own home (Schirilla, 2015). Alternative designs for LTCI remain under discussion but long‐term future perspectives and responsible handling of this problem are still missing. Instead, LTHC in Germany relies on ICs and/or limited cash support for CRs, which increases irregular and (il)legal migrant care workers (Kniejska, 2016).

If the present situation is evaluated in the light of the empirical findings reported here and with regard to existing normative standards concerning participation and inclusion (e.g. Article 3 (c) Convention on the Rights of Persons with Disabilities, Article 4 and 6 Charter of Rights for People in Need of Long Term Care and Assistance (German Federal Ministry of Family Affairs Senior Citizens Women and Youth & German Federal Ministry of Health, 2007), Article 1 and 2 German Social Security Code XI), it has to be concluded that the actual situation does not meet the self‐given societal goals. The ambivalent impact of home care on social participation (Dahlberg & McKee, 2016) remains widely unrecognized: for those living alone, professional home nursing enables social participation for CRs (again), but professional home nursing is restricting the social participation and privacy of ICs. Loneliness and its adverse effects on health become increasingly important and receive more and more attention (Gerst‐Emerson & Jayawardhana, 2015), but Germany has to make up leeway.

Interestingly, the perceptions regarding the statutory benefits differed greatly between the groups: CRs and ICs thought that they did not get what they needed but instead services they do not need. However, this view was not shared by the PCs who are familiar with the system. They saw it less problematic and as a luxury one can afford if saving money. Against the background of the growing number of frail older people living in low‐income households and poverty (Stolz, Mayerl, Waxenegger, & Freidl, 2017)—which counts for current CRs as well as for individuals that give or gave informal care—questions concerning shortages in the context of increasing social inequality in Germany should be re‐discussed (Kümpers & Alisch, 2018). As social participation is most important for CRs, it appears to be obvious that the serious impact of LTHC on caregivers can be outweighed by the gain of constant interpersonal interaction for the CR. This, however, can be a fallacy, as the phenomenon of “existential loneliness” occurs even in such arrangements and that its appearance is judged differently by CRs and their ICs and PCs (Larsson, Edberg, Bolmsjo, & Ramgard, 2018; Sundstrom, Edberg, Ramgard, & Blomqvist, 2018).

The study's methodological key strength is the openness which creates narratives that allow an in‐depth description of the current practices. However, study limitations arise from the sample size and the missing generalizability of the results that are well‐known limitations in qualitative research. Our sample is homogeneous with regard to age and socio‐cultural background. Further research should thus build a sample with participants from different ethnicities and age. In addition, the nature of the disease(s) which are underlying cause(s) for care dependency might also influence the specific needs and should thus be part of a purposive sampling.

## 6 CONCLUSIONS

It can be summarized that the present problem‐solving strategies for the shortcomings of LTCI reveal social inequality in LTHC arrangements. Thus, the results can be used to support the practitioners with the identification of individuals at the greatest risk of over‐, under‐ and misprovision. The results presented can serve as a basis for designing a quantitative survey on the effects of the latest LTCI reform, evaluating specifically the domains of over‐, under‐ and misprovision described.

Future problems concerning the supply and organization of LTHC in arrangements combining informal and formal care remain urgent and unresolved; a task that politics should attend to in further advancement of the LTCI based on such cross‐sectional studies.

## CONFLICT OF INTEREST

All authors (AHS, MB, SS, IB) declare that they have no financial or non‐financial competing interests. All authors declare that they have no financial relationships that might be perceived as a potential conflict of interest.

## AUTHOR CONTRIBUTIONS

All authors (AHS, MB, SS and IB) analysed and interpreted the data using qualitative methods. AHS, SS and IB conceptualized the paper and contributed essentially to the writing of this manuscript. AHS, MB, SS and IB read and approved the final manuscript.

## ETHICAL APPROVAL

The study was approved by the institutional review board of the University Medicine Greifswald (reference number BB123/16). Written informed consent was elicited from each study participant.

## Supporting information

Appendix S1

Appendix S2
