# Supplementary material for: Adequacy of care provision in long‐term home nursing arrangements: A triangulation of three perspectives
Source: Nurs Open. 2020 Jul 2;7(5):1634–42. doi: 10.1002/nop2.548 (PMC7424429; doi:10.1002/nop2.548)
Supplement: Supplementary file 1 — Appendix S1 [file NOP2-7-1634-s001.docx]

**Appendix S1: Relevant reforms concerning Germany’s long-term home care sector**

Two recent health care reforms are particularly important for the interpretation of the study results against the background of the German health care system: the “Second Bill to Strengthen Long-Term Care” (Table I) and the “New Nursing Profession Law” (Table II).

| **Second Bill to Strengthen Long-Term Care (PSG II)** | | |
| --- | --- | --- |
| Various reforms of the LTC insurance aimed to enhance this fifth pillar of social security (Bäcker, 2016; Nadash, Doty, & von Schwanenflügel, 2017; Ranci & Pavolini, 2015). The Second Bill to Strengthen Long-Term Care (PSG II) is one of three “Bills to Strengthen Long-Term Care” (Federal Ministry of Health, 2016). The PSG II, which came into power in 2017, represents the most far-reaching changes since the implementation of the LTC insurance in Germany in 1995. PSG II introduced a new definition of LTC dependency and a new assessment instrument to determine care dependency (Medizinischer Dienst des Spitzenverbandes Bund der Krankenkassen e.V., 2017). | | |
|  | previous definition and assessment of care dependency | new (current) definition and assessment of care dependency |
| - Care dependents | were (old law) “individuals who require substantial or greater assistance for the ordinary and regularly recurring activities of life in the long term because of a physical, mental or emotional disease or disability”^1^ | are “individuals who are in need of assistance through others due to health-related impairments of self-reliance or abilities” (§14 para.1 German Code of Social Law XI)^1^ |
| - Assessment | - mainly physical constraints  - assessment through time needed  - three Levels of Care | - mental restrictions equal to physical constraints  - six aspects of life are weighted and considered (e.g. mobility, cognitive abilities)  - five Care Grades |

Table I: Second Bill to Strengthen Long-Term Care (PSG II), ^1^ translated by the authors, LTC long-term care

| **New Nursing Profession Law (PflBRefG)** | |
| --- | --- |
| Compared to other European countries, Germany not only differs by the less attractive working and employment conditions but also by the low share of academisation (Dreier, Homeyer, Oppermann, Hingst, & Hoffmann, 2016; Wissenschaftsrat, 2012) and the fundamental differences in the vocational training for different nursing specialisations (Bundesminsterium für Bildung und Forschung, 2014, p. 143ff.; Lahtinen, Leino-Kilpi, & Salminen, 2014).  The PflBRefG aims – amongst others – to integrate the three existing specialisations to increase attractiveness and international connectivity. It came into power in 2017 and was introduced on January 1^st^ 2020. | |
| former „Geriatric Nursing Law“ and „Nursing Law” | new “Nursing Profession Law” |
| - 3 years vocational training | |
| - three different vocational training programmes for three different nursing specialisations (“General Nursing”, “Paediatric Nursing”, “Geriatric Nursing”) - different curricula regulated by the law separately - Geriatric Nursing students pay school fees | - 2 years: joint, generalist education in which they choose an area of specialisation in practical training - trainees who choose to continue generalist in the third year of training acquire the vocational qualification and title “nursing specialist” („Pflegefachfrau/-mann“) - trainees who focus on nursing for elderly, children or adolescents can choose whether they want to acquire a separate qualification instead of continuing generalist education - additional nursing studies at the university are possible |

Table II: New Nursing Profession Law (PflBRefG)

**References**

Bäcker, G. (2016). Reform of the long-term care insurance in Germany. Retrieved from <http://ec.europa.eu/social/BlobServlet?docId=16074&langId=en>

Bundesminsterium für Bildung und Forschung. (2014). Bestandsaufnahme der Ausbildung in den Gesundheitsfachberufen im europäischen Vergleich. Retrieved from <https://www.bmbf.de/upload_filestore/pub/berufsbildungsforschung_band_15.pdf>

Dreier, A., Homeyer, S., Oppermann, R., Hingst, P., & Hoffmann, W. (2016). Akademische Pflegeausbildung in Deutschland: Ergebnisse zur pflegefachlichen Weiterentwicklung aus der Care-N Study M-V. *Zeitschrift für Evidenz, Fortbildildung und Qualität iml Gesundhwesen, 115-116*, 63-70.

Federal Ministry of Health. (2016). Germany's Long Term Care Strengthening Acts. Retrieved from [https://www.bundesgesundheitsministerium.de/en/topics/long-term-care/germanys-long-term-care-strengthening-acts.html - c4262](https://www.bundesgesundheitsministerium.de/en/topics/long-term-care/germanys-long-term-care-strengthening-acts.html#c4262)

Lahtinen, P., Leino-Kilpi, H., & Salminen, L. (2014). Nursing education in the European higher education area — Variations in implementation. *Nurse Education Today, 34*(6), 1040–1047. doi:10.1016/j.nedt.2013.09.011

Medizinischer Dienst des Spitzenverbandes Bund der Krankenkassen e.V. (2017). Richtlinien des GKV-Spitzenverbandes zur Feststellung der Pflegebedürftigkeit nach dem XI. Buch des Sozialgesetzbuches. Retrieved from <https://www.mds-ev.de/fileadmin/dokumente/Publikationen/SPV/Begutachtungsgrundlagen/17-07-17_BRi_Pflege.pdf>

Nadash, P., Doty, P., & von Schwanenflügel, M. (2017). The German Long-Term Care Insurance Program: Evolution and Recent Developments. *The Gerontologist, 57*(5).

Ranci, C., & Pavolini, E. (2015). Not all that glitters is gold: Long-term care reforms in the last two decades in Europe. *Journal of European Social Policy, 25*(3), 270-285.

Wissenschaftsrat. (2012). Recommendations on higher education qualifications for the healthcare system. Retrieved from <https://www.wissenschaftsrat.de/download/archiv/2411-12_Executive-Summary.pdf>
