# Supplementary material for: Adequacy of care provision in long‐term home nursing arrangements: A triangulation of three perspectives
Source: Nurs Open. 2020 Jul 2;7(5):1634–42. doi: 10.1002/nop2.548 (PMC7424429; doi:10.1002/nop2.548)
Supplement: Supplementary file 2 — Appendix S2 [file NOP2-7-1634-s002.docx]

| **Key Questions** | **Themes** |
| --- | --- |
| In which way do you support your relative (the care recipient) in everyday life? | Care situation |
| Do you think that overall your relative (the care recipient) is getting enough support? | Adequacy of care provision |
| Are there aspects where you feel there should be more support for him/her (the care recipient)? | Underprovision of care |
| Is there a “too much” of supply/support at any point? | Overprovision of care |
| What do you think about the current system of classification, i.e. Care Levels? Do you think that your relative has the correct/suitable Care Level?  Are there any problems? | Care Levels |
| What advice would you give to someone who is caring for a relative? In your view, what is particularly important for good care within the family? | Role switch: Nursing service |
| Finally, I would like to do a thought experiment with you: Please imagine that you were in the situation of the care recipient. What would be particularly important to you for good home care? | Role switch: Care recipient |

Table 1: Excerpt interview guide for informal caregivers

| **Key Questions** | **Themes** |
| --- | --- |
| Who supports you in everyday life?  With what? | Care situation |
| Is this assistance (enough) for you?  If not, why not? / If yes: Do you feel well taken care of overall? | Adequacy of care provision  Underprovision of care |
| Are there some tasks that you are currently getting help with, but which you could still do on your own?  Do you sometimes feel “too much” taken care of? (If so: Can you describe this to me in more detail? If difficult: Can you perhaps explain this to me using an example?) | Overprovision of care |
| Imagine working in a home nursing service: Would you do anything differently?  If not, why not? If yes, why? (What would you do differently? Can you give me some examples) | Role switch: Professional caregiver |

Table 2: Excerpt interview guide for care recipients

| **Key Questions** | **Themes** |
| --- | --- |
| In which way do you support the care recipients predominantly in everyday life? | Care situation |
| Is this assistance enough?  Are there certain areas/tasks of which you think there should be more support? | Adequacy of care provision  Underprovision of care |
| Is there a „too much“ of care at any point? | Overprovision of care |
| What do you think about the current system of classification, i.e. Care Levels? Do you think that the care recipients are classified correctly?  Are there any problems? | Care Levels |
| Finally, I would like to do a thought experiment with you: Please imagine that you were in the situation of the care recipient. What would be particularly important to you for good home care? | Role Switch: Care recipient |

Table 3: Excerpt interview guide for professional caregivers
